# Effect of phenylephrine versus ephedrine on the incidence of postoperative delirium in elderly patients undergoing non-cardiac and non-neurosurgical procedures: protocol for a multicentre, double-blind, randomized, controlled trial

**DOI:** 10.1080/07853890.2026.2732555

**Published:** 2026-09-16

**Authors:** Xiaoxiao Xu, Gang Wang, Xinyu Li, Ke Peng, Hong Liu, Fuhai Ji, Xisheng Shan, Yulan Wang

**Affiliations:** ^a^ Department of Anesthesiology, First Affiliated Hospital of Soochow University, Suzhou, Jiangsu, China; ^b^ Institute of Anesthesiology, Soochow University, Suzhou, Jiangsu, China; ^c^ Department of Anesthesiology, The Fifth People’s Hospital of Huai’an, Huai’an, Jiangsu, China; ^d^ Department of Anesthesiology and Pain Medicine, University of California Davis Health, Sacramento, CA, USA

**Keywords:** Postoperative delirium, ephedrine, phenylephrine, general anaesthesia, elderly

## Abstract

**Background:**

Whether ephedrine, when used to maintain intraoperative blood pressure, is superior to phenylephrine in reducing postoperative delirium remains uncertain. This study aims to determine whether the intraoperative management of hypotension with ephedrine, compared with phenylephrine, reduces the incidence of postoperative delirium in elderly patients undergoing major non-cardiac, non-neurosurgical surgery.

**Methods:**

This multicentre, randomized, double-blind, controlled trial will enrol 1,084 elderly patients scheduled for elective non-cardiac and non-neurosurgical major surgeries under general anaesthesia at seven hospitals in China. Participants will be randomized in a 1:1 ratio, stratified by study centre, to receive continuous intravenous infusion of either phenylephrine or ephedrine to maintain mean arterial pressure within 20% of the baseline value. The primary outcome is the incidence of postoperative delirium within 7 days after surgery. Secondary outcomes include severity of postoperative delirium, numeric rating scale pain scores at rest and during activity at 24, 48 and 72 h postoperatively, intensive care unit (ICU) admission rate and length of ICU stay, length of postoperative hospital stay, incidence of in-hospital major adverse cardiovascular and cerebrovascular events (myocardial infarction, arrhythmia, heart failure, and stroke), and 30-day all-cause mortality.

**Discussion:**

We hypothesize that ephedrine, when used to maintain intraoperative blood pressure, is superior to phenylephrine in reducing postoperative delirium in elderly patients undergoing major non-cardiac, non-neurosurgical surgery. The findings of this study may provide high-quality evidence for optimizing the management of intraoperative hypotension in this vulnerable population.

**Trial registration:**

Chinese Clinical Trial Registry (ChiCTR2500115385).

## Background

Postoperative delirium (POD) is an acute neuropsychiatric syndrome characterized by fluctuating disturbances in attention, awareness, and cognition [1]. The incidence of POD in elderly surgical patients ranges from 10% to 47%, varying across surgical procedures [2]. POD significantly impairs postoperative recovery, leading to prolonged hospital stays, increased complication rates, elevated healthcare costs, and higher mortality [1]. Although the aetiology of POD is multifactorial, cerebral hypoperfusion and impaired cerebral oxygenation are regarded as pivotal pathophysiological contributors [3,4]. Intraoperative hypotension may compromise cerebral blood flow, disrupt metabolic homeostasis, and induce neuronal injury, thereby increasing the susceptibility to delirium [3,5].

Vasoactive medications are routinely administered to prevent and treat intraoperative hypotension during general anaesthesia, with phenylephrine and ephedrine representing the most commonly utilized agents. Emerging evidence suggests that their distinct hemodynamic profiles may differentially affect cerebral perfusion. Ephedrine, a mixed α- and β-adrenergic agonist, augments cardiac output primarily by increasing myocardial contractility and heart rate [6,7]. In contrast, phenylephrine, a selective α_1_-adrenergic agonist, elevates blood pressure *via* peripheral vasoconstriction but may concurrently reduce cardiac output and induce cerebral vasoconstriction—effects that could compromise cerebral oxygen delivery [8,9]. A recent study demonstrated that, compared with phenylephrine, ephedrine administration was associated with increased cerebral blood flow and higher regional cerebral oxygen saturation in the normal contralateral hemisphere of anaesthetized patients undergoing brain tumour resection [10]. Such alterations in cerebral haemodynamics may contribute to differences in the incidence of postoperative delirium.

However, clinical consensus regarding the comparative impact of these agents on neurological outcomes remains elusive. A retrospective cohort study of adult patients undergoing general anaesthesia for non-cardiac, non-neurosurgical procedures reported that intraoperative administration of ephedrine, compared with phenylephrine, was associated with lower odds of postoperative delirium [11]. In contrast, a randomized trial suggested that the beneficial effect of ephedrine on delirium incidence may be temporally limited, with a reduction observed only on postoperative day 1 and comparable rates between groups on subsequent days [12]. These inconsistent findings, derived from studies with limited sample sizes and heterogeneous designs, underscore the need for a multicentre trial to clarify the influence of these two vasoactive agents on postoperative delirium.

Accordingly, we propose a multicentre, double-blind, randomized controlled trial to test the hypothesis that ephedrine, when used to maintain intraoperative blood pressure, is superior to phenylephrine in reducing postoperative delirium in elderly patients undergoing major non-cardiac, non-neurosurgical surgery.

## Methods

### Ethics and registration

The study protocol has been approved by the Institutional Review Board of the leading centre (the First Affiliated Hospital of Soochow University, Suzhou; Approval No. 2025-441-1) and the ethics committees of all participating centres (Affiliated Hospital of Jiangsu University [Approval No. KY2025H0926-08], Taicang First People’s Hospital [Approval No. 2025-SR-098], Wuxi People’s Hospital [Approval No. KY25188], Affiliated Hospital of Xuzhou Medical University [Approval No. XYFY2025-KL311-01], The First Affiliated Hospital of University of Science and Technology of China [Approval No. 2025KY-414], Renmin Hospital of Wuhan University [Approval No. WDRY2026-K002]). This trial was prospectively registered on the Chinese Clinical Trial Registry (http://www.chictr.org.cn; identifier: [ChiCTR2500115385]) on 25 December 2025, before the enrolment of the first participant.

### Study design and participants

This investigator-initiated, multicentre, double-blind, randomized, controlled trial is being conducted across seven tertiary hospitals in China. Participant recruitment commenced on 30 December 2025, with study completion anticipated by December 2027. All participants will provide written informed consent prior to enrolment. A total of 1,084 eligible patients will be randomly assigned in a 1:1 ratio to receive either phenylephrine or ephedrine (542 patients per group, Figure 1). This study will be conducted in accordance with the principles of the Declaration of Helsinki. The schedule of enrolment, interventions, and outcome assessments is presented in Table 1, in accordance with the Standard Protocol Items: Recommendations for Interventional Trials (SPIRIT) statement (Supplement 1).

**Figure 1.**
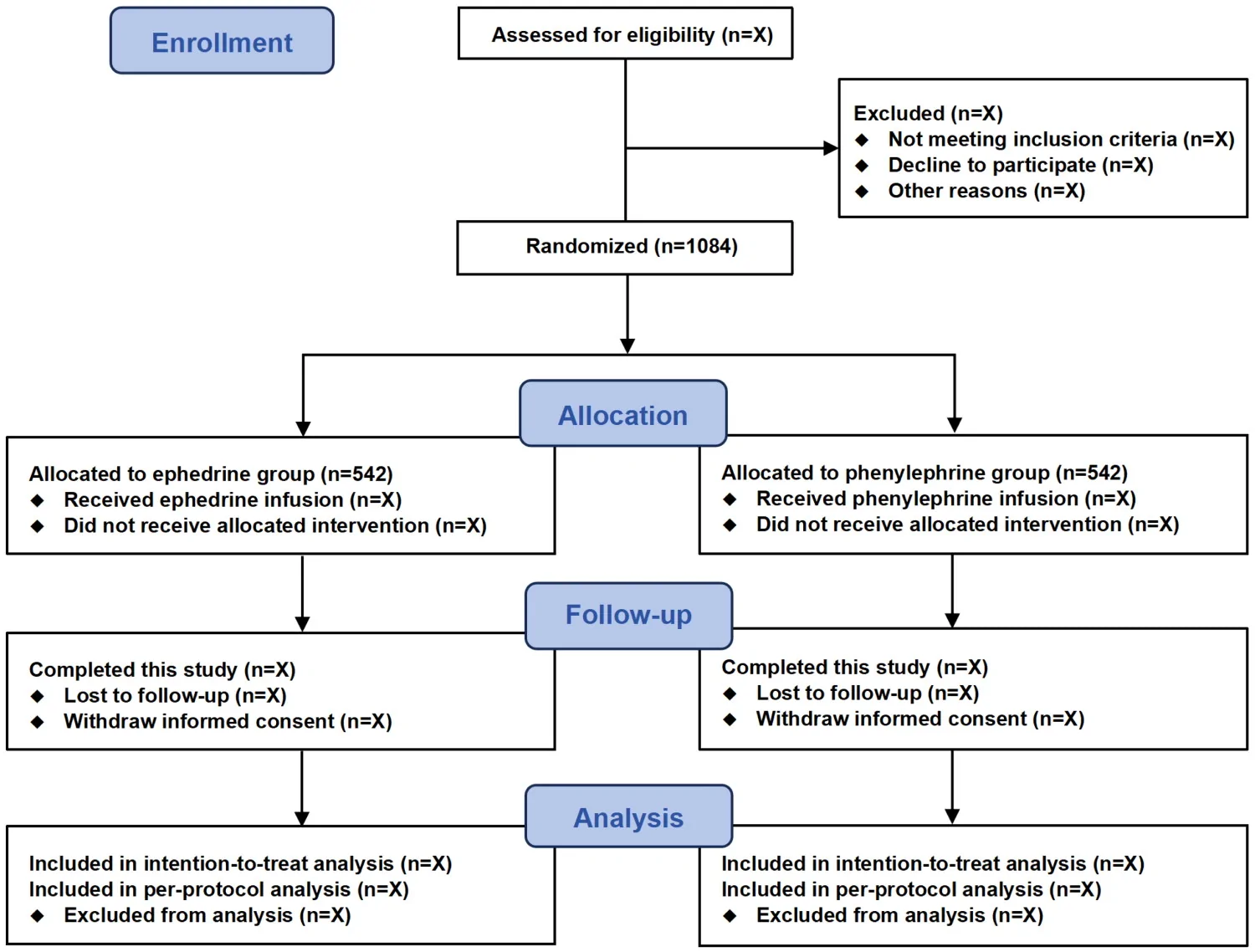
Study flowchart.

**Table 1.** Schedule of patient enrolment, intraoperative data collection, and outcome measurements.

	Study period							
Time point	Enrolment	Allocation	Post-allocation					Close-out
	Preoperative visit	Prior to surgery	During surgery	PACU	Postoperative days 1–3	Postoperative days 4–7	Hospital discharge	Postoperative day 30
**Enrolment**								
Eligibility screening	**╳**							
Written informed consent	**╳**							
Demographic data	**╳**							
Baseline characteristics	**╳**							
MMSE	**╳**							
Delirium	**╳**							
Randomization		**╳**						
Allocation		**╳**						
**Interventions**								
Ephedrine			**╳**					
Phenylephrine			**╳**					
**Measurements**								
Intraoperative information			**╳**					
Extubation time			**╳**	**╳**				
Length of PACU stay				**╳**				
POD					**╳**	**╳**		
Severity of POD					**╳**	**╳**		
NRS pain scores					**╳**			
ICU admission					**╳**	**╳**	**╳**	
Length of ICU stay					**╳**	**╳**	**╳**	
Postoperative hospital stay							**╳**	
Adverse cardiovascular and cerebrovascular events^a^					**╳**	**╳**	**╳**	
30-day all-cause mortality								**╳**

Abbreviations: ICU, Intensive Care Unit; MMSE: Mini-Mental State Examination; NRS: Numeric Rating Scale; PACU: Post-Anaesthesia Care Unit; POD: Postoperative Delirium.

^a^Including myocardial infarction, arrhythmia, heart failure, and stroke.

Eligible participants included: (1) age ≥ 65 years, (2) American Society of Anesthesiologists (ASA) physical status I─III, (3) scheduled for elective major non-cardiac, non-neurosurgical surgery under general anaesthesia, and (4) an expected surgical duration > 2 h.

Exclusion Criteria were as follows: (1) planned intraoperative use of other vasoactive agents (e.g. norepinephrine, dopamine, epinephrine), (2) a history of stroke, Parkinson’s disease, or epilepsy, (3) language barriers, or significant hearing or visual impairment precluding effective communication, (4) a history of psychiatric disorders (e.g. depression, generalized anxiety disorder, schizophrenia), (5) severe cardiovascular disease (New York Heart Association function class ≥ III), (6) severe hepatic dysfunction (Child-Pugh Class C), (7) renal failure requiring renal replacement therapy, (8) a history of alcohol or substance abuse, psychological disorders, or anticipated non-adherence, and (9) concurrent participation in another clinical trial.

### Randomization and blinding

An independent statistician, not involved in trial implementation, will generate the randomization sequence using SAS statistical software (version 9.4, SAS Institute Inc.). Participants will be randomized in a 1:1 ratio with permuted block size of 4, stratified by study centre. The allocation assignments will be sealed in sequentially numbered, opaque envelopes, which are stored in a locked cabinet. An independent research coordinator, with no role in patient management or outcome assessment, will open the envelopes to prepare the allocated study medication, and label the syringes with patient identification number. Both study solutions (phenylephrine and ephedrine) are colourless and transparent, and therefore visually indistinguishable. Two independent, blinded investigators will perform postoperative follow-up and delirium assessments. Patients, perioperative care providers (surgeons, anaesthesiologists, and nurses), and outcome assessors will remain blinded to treatment allocation throughout the study.

### Anaesthesia and perioperative management

All patients will follow an 8-hour preoperative fast, with clear fluids allowed until 2 h before anaesthesia induction. No preoperative medications will be administered to either group. Upon arrival in the operating room, standard monitoring-including electrocardiogram, pulse oximetry, and non-invasive blood pressure-will be initiated. After local anaesthesia, invasive arterial blood pressure monitoring will be established *via* radial artery catheterization. Anaesthesia will be induced according to a standardized protocol involving the sequential intravenous administration of sufentanil (0.4 µg/kg), propofol (1–2 mg/kg), and a neuromuscular blocking agent (cisatracurium 0.2 mg/kg or rocuronium 1 mg/kg). Endotracheal intubation will then be performed, and mechanical ventilation will be adjusted to maintain end-tidal carbon dioxide (PETCO_2_) at 35–45 mmHg.

General anaesthesia will be maintained with sevoflurane (1-3%), titrated to achieve bispectral index value between 40 and 60. Analgesia will be provided by infusion of remifentanil (0.1–0.3 µg/kg/min), with supplemental boluses of sufentanil (0.1–0.2 µg/kg) administered as clinically indicated. Neuromuscular blockade will be maintained using a continuous infusion of either cisatracurium (1–2 µg/kg/min) or rocuronium (5–6 µg/kg/min), consistent with the agent used for induction. Lactated Ringer’s solution was used as the default intraoperative crystalloid to maintain fluid balance, with the infusion rate of 5–7 mL/kg/h. Colloids (gelatin or hydroxyethyl starch) will be administered according to the anaesthesiologist’s assessment of intraoperative fluid loss. Red blood cell transfusion will be performed if the haemoglobin concentration falls below 7 g/dL. Any medications known to influence cognitive function, including scopolamine, midazolam, or dexmedetomidine, are not permitted during the study period. 5-HT_3_ receptor antagonists (ondansetron, granisetron, or tropisetron) will be administered for antiemesis. Active warming will be provided using a forced-air warming blanket to maintain nasopharyngeal temperature between 36 and 37 °C. Postoperative analgesia will follow the standard practice of each participating center, with all medications recorded in the case report form (CRF). Upon completion of surgery, patients will be transferred to the post-anaesthesia care unit (PACU) and subsequently to the surgical floor. Discharge from the PACU will require a modified Aldrete Score ≥ 9.

### Study intervention and perioperative hemodynamic management

Hemodynamic stability will be supported using a continuous infusion of either phenylephrine (0.1 mg/mL) or ephedrine (2 mg/mL) according to the allocated group. The dosing regimens for ephedrine and phenylephrine infusion are selected based on established infusion schemes from prior studies and clinical recommendations [10,13,14]. The preoperative baseline MAP is defined as the average of three non-invasive blood pressure measurements taken during the preoperative visit on the day before surgery. Intraoperative hypotension is defined as mean artery pressure (MAP) below 20% of the preoperative baseline value or an absolute value of 65 mmHg. The designated vasoactive infusion will commence at anaesthesia induction and continued until skin closure. The infusion rate will initiate at 10–30 ml/h, with the rate titrated to maintain the patient’s MAP within 20% of the preoperative baseline or at least 65 mmHg. If hypotension persists for more than 3 min despite maximal titration of the assigned study drug, the attending anaesthesiologist may implement alternative hemodynamic management strategies at their clinical discretion [15]. Any deviation from the assigned infusion must be comprehensively documented in the CRF. Hypertension, defined as a MAP exceeding 20% above the baseline, the infusion of vasopressor agents will be stopped. If hypertension persists following cessation, pharmacologic intervention with agents such as nicardipine, nitroglycerin, or urapidil will be initiated. Bradycardia, defined as a heart rate of less than 45 beats per minute, will be treated with atropine. Tachycardia, defined as a heart rate greater than 100 beats per minute, will be treated with esmolol.

### Study outcomes

The primary outcome is the incidence of postoperative delirium within the first 7 postoperative days. Delirium will be assessed daily by trained assessors blinded to group allocation using the Confusion Assessment Method (CAM), supplemented with a proximate look-back approach involving extraction of relevant medical and nursing documentation from the preceding 24 h [16]. For patients admitted to the intensive care unit (ICU) postoperatively, delirium will be evaluated using the CAM-ICU [17]. Secondary outcomes include the severity of postoperative delirium, measured using the CAM-Severity (CAM-S) scale [18]; numeric rating scale (NRS) pain scores (at rest and during activity) at 24, 48 and 72 h postoperatively; ICU admission and ICU length of stay; length of postoperative hospital stay; incidence of in-hospital major adverse cardiovascular and cerebrovascular events (myocardial infarction, arrhythmia, heart failure, and stroke); and 30-day all-cause mortality.

### Data collection

Demographic data, baseline characteristics, and study outcomes will be collected by trained, independent investigators who are blinded to group allocation. Demographic and baseline variables will include age, sex, weight, height, body mass index (BMI), ASA physical status, medical and surgical history, preoperative medication use, preoperative cognitive function assessed using the Mini-Mental State Examination (MMSE), preoperative delirium status, preoperative blood pressure and heart rate, and laboratory test. Perioperative variables will include anaesthetic exposure (e.g. doses of propofol, sufentanil, remifentanil, and sevoflurane), type and duration of surgery, intraoperative hemodynamic parameters, fluid balance, blood loss, urine output, transfusion, hemodynamic adverse events, extubation time, length of PACU, ICU admission and ICU length of stay.

Delirium will be assessed preoperatively and then daily between 16:00 and 20:00 postoperatively through face-to-face evaluations using either CAM or CAM-ICU for patients in the ICU. The CAM evaluates for core features: (1) acute onset and fluctuating course; (2) inattention; (3) disorganized thinking; and (4) altered levels of consciousness. The CAM is considered to be positive for the presence of delirium if both features 1 and 2 are present, with at least one of features 3 or 4 [16]. For patients discharged before postoperative day 7, a structured telephone assessment comprising a modified telephone MMSE and the delirium symptom interview will be conducted after discharge until postoperative day 7, and delirium will be determined using the CAM diagnostic algorithm [19]. Before performing study assessments, all investigators responsible for CAM or CAM-ICU evaluations will undergo standardized training in administration and scoring. Standardized training materials and calibration exercises using clinical scenarios will be used across participating centres to promote consistency in delirium assessment. Major adverse cardiovascular and cerebrovascular events will be monitored throughout the hospitalization period. The definitions of adverse events are based on the Common Terminology Criteria for Adverse Events (Supplement 2, Table S1). All data will be collected using the CRF and will be entered in the electronic data capture system within 1 week of completing the CRF forms. An independent data safety and monitoring committee (DSMC) will oversee trial conduct to ensure participant safety and maintain the integrity and quality of the study data.

### Sample size calculation

Based on previous literature, the incidence of POD in elderly patients undergoing non-cardiac surgery is estimated to be approximately 19.0% [20]. We hypothesize that ephedrine treatment only will reduce the POD incidence by at least one-third, corresponding to a relative risk reduction of 34%, and an expected incidence of 12.5% in the ephedrine group [11]. Assuming a two-sided significance level (α) of 0.05 and 80% power (1-β), a sample size of 492 patients per group is required. Accounting for an estimated dropout rate of 10%, we plan to enroll 542 patients per group, for a total of 1084 patients (PASS version 15.0.5, PASS Institute Inc.).

### Statistical analysis

Categorical data will be presented as frequencies (percentages) and analyzed using Chi-square tests or Fisher’s exact test, as appropriate. Continuous variables will be tested for normality using the Shapiro-Wilk test. Normally distributed data will be presented as mean ± standard deviation (SD) and compared using independent samples t-tests, whereas non-normally distributed data will be presented as median (interquartile range [IQR]) and compared using the Mann-Whitney U test. Effect estimates will be reported as relative risks (RR) for categorical outcomes or as mean/median differences for continuous outcomes, each with corresponding 95% confidence intervals (CIs).

For study outcomes, mixed-effects regression models will be applied for adjusted analyses. The model will include fixed effects for age, sex, preoperative dementia, preoperative delirium, and treatment group, with recruitment site specified as random effects. Prespecified subgroup analysis will be performed according to patient age (65–80 years vs. >80 years), sex, preoperative cognitive impairment/dementia (yes vs. no), preoperative delirium (yes vs. no), and study centre (leading vs. participating).

The primary analysis will follow the intention-to-treat principle, including all randomized patients who undergo surgery and have primary outcome data available, analyzed according to their assigned treatment group. The per-protocol population will exclude patients with major protocol deviations, those undergoing reoperation or other surgical procedures within the first 7 postoperative days, and those receiving non-assigned vasoactive agents, including patients requiring rescue vasoactive therapy for persistent hypotension. After enrolment of 50% of the planned sample size, an interim analysis will be conducted by an independent statistician with access to unblinded treatment assignments. The analysis will include assessment of efficacy using an O’Brien–Fleming boundary, conditional power, and safety outcomes. The interim results will be reviewed by the independent DSMC, which will be authorized to determine whether the trial should proceed or be terminated early.

Missing primary outcome data are expected to be minimal (< 5%). No imputation will be performed for the primary analysis. To assess the robustness of the findings to missing primary outcome data, a sensitivity analysis using multiple imputation by chained equations will be performed. Several perioperative variables potentially associated with postoperative delirium, including perioperative hypotension, blood transfusion, analgesic technique, postoperative opioid exposure, and postoperative ICU admission, will be adjusted for in exploratory sensitivity analyses. All statistical tests will be two-sided, with statistical significance defined as *p* < 0.05. Statistical analyses will be conducted using SAS software (version 9.4, SAS Institute Inc.) and R software (version 4.3.0; R Foundation for Statistical Computing).

## Discussion

This multicentre, randomized, double-blind, clinical trial will enrol 1,084 eligible patients to evaluate the effects of intraoperative phenylephrine versus ephedrine infusion on the incidence of postoperative delirium during major non-cardiac, non-neurosurgical surgery. In addition, we will assess a range of patient-centred outcomes, including delirium severity, postoperative pain intensity, resource utilization (ICU admission and postoperative hospital stay), postoperative complications, and mortality. The trial will be conducted and reported in accordance with the Consolidated Standards of Reporting Trials guidelines.

POD is a common and clinically significant complication in the elderly, with reported incidence rates ranging from 10% to 47% depending on the surgical population [1,21,22]. POD is associated with prolonged hospitalization, increased healthcare costs, higher postoperative morbidity and mortality, and an elevated risk of long-term cognitive decline and dementia [1,23]. Although its pathogenesis is multifactorial, intraoperative hypotension is widely recognized as an important and modifiable anaesthesia-related risk factor [3]. Hypotension can compromise tissue perfusion and oxygen delivery to vital organs, including the brain [15]. Furthermore, age-related reductions in cerebrovascular reserve may limit compensatory capacity during hemodynamic fluctuations, thereby increasing susceptibility to cerebral ischemia and subsequent neurocognitive dysfunction, including delirium [24–26].

Thus, the choice of vasopressor may be clinically important. Ephedrine increases blood pressure primarily by promoting the release of norepinephrine and epinephrine from sympathetic nerve terminals, thereby activating α1-adrenergic receptors to induce vasoconstriction and β1-adrenergic receptors to enhance cardiac contractility and heart rate, ultimately increasing cardiac output [6,7]. While phenylephrine raises blood pressure predominantly by increasing systemic vascular resistance through selective α1-adrenergic–mediated peripheral arterial vasoconstriction, this increase in afterload may be accompanied by a reduction in cardiac output [27,28]. In a study of anaesthetized patients undergoing brain tumour surgery, ephedrine was shown to increase both cerebral blood flow and the cerebral metabolic rate of oxygen in the contralateral healthy hemisphere, whereas phenylephrine decreased these parameters [10]. Neuroimaging studies have further suggested that although both agents effectively raise mean blood pressure, ephedrine may confer greater improvements in global and regional cerebral blood flow and tissue oxygenation compared with phenylephrine [8,13]. Moreover, ephedrine’s ability to cross the blood–brain barrier distinguishes it from other vasopressors, such as phenylephrine and norepinephrine, potentially enabling direct effects on central nervous system targets [6,29]. These findings suggest that ephedrine may offer neuroprotective benefits, particularly in high-risk populations such as older adults or individuals with preexisting cognitive or cerebrovascular vulnerabilities.

A recent multicentre retrospective cohort study of 103,094 hospitalized adults undergoing general anaesthesia for non-cardiac, non-neurosurgical procedures reported that intraoperative administration of ephedrine, compared with phenylephrine, was associated with lower odds of postoperative delirium. In addition, a prospective randomized study including 142 older adults undergoing elective total hip or knee arthroplasty found that ephedrine used to treat intraoperative hypotension was associated with a lower incidence of POD within the first three postoperative days compared with phenylephrine. However, the available evidence remains inconclusive. Another randomized trial suggested that ephedrine reduced POD incidence only during the early postoperative period, whereas rates were comparable between groups on postoperative days 2–3. To our knowledge, the present study will be the first multicentre, randomized, double-blind trial to evaluate whether ephedrine, compared with phenylephrine, reduces the incidence of POD in older adults undergoing non-cardiac, non-neurosurgical surgery. This study protocol administers the study drugs *via* continuous infusion, an approach supported by existing clinical evidence and practice guidelines to achieve more stable hemodynamic control [30,31].

This study has several limitations. First, delirium assessments will be performed once daily, which may miss episodes of fluctuating delirium, particularly hypoactive subtypes. To address this limitation, CAM evaluations will be supplemented with a systematic review of medical and nursing records for documented symptoms or clinical features suggestive of delirium. Second, infusion of phenylephrine and ephedrine may differentially affect heart rate, potentially providing cues that could compromise blinding. Third, for patients discharged before completion of the 7-day follow-up period, delirium will be assessed by telephone, which may be less sensitive than face-to-face evaluation. However, a structured telephone interview based on the CAM diagnostic algorithm will be used, an approach that has previously been validated against face-to-face delirium assessment.

In conclusion, this multicentre randomized controlled trial is designed to determine whether ephedrine, used to maintain intraoperative blood pressure, is superior to phenylephrine in reducing postoperative delirium in elderly patients undergoing major non-cardiac, non-neurosurgical surgery. The results are expected to provide high-quality evidence to guide optimal management of intraoperative hypotension in this vulnerable population.

## Supplementary Material

Supplement 2.DOCX

Supplement 1.doc

## Data Availability

Data sharing is not applicable to this article as no data were created or analyzed in this study.
