# Supplementary material for: Effect of phenylephrine versus ephedrine on the incidence of postoperative delirium in elderly patients undergoing non-cardiac and non-neurosurgical procedures: protocol for a multicentre, double-blind, randomized, controlled trial
Source: Ann Med. 2026 Sep 16;58(1):2732555. doi: 10.1080/07853890.2026.2732555 (PMC13617960; doi:10.1080/07853890.2026.2732555)
Supplement: Supplementary file 1 [file IANN_A_2732555_SM6815.docx]

**Table S1. Definitions of adverse events during hospitalization**

| **Event** | **Definition** |
| --- | --- |
| Hypotension | hypotension is defined as mean artery pressure (MAP) below 20% of the preoperative baseline value or an absolute value of 65 mmHg. |
| Hypertension | Hypertension, defined as a MAP exceeding 20% above the baseline value. |
| Bradycardia | Bradycardia, defined as a heart rate of less than 45 beats per minute. |
| Tachycardia | Tachycardia, defined as a heart rate greater than 100 beats per minute. |
| Myocardial infarction | Myocardial infarction was defined as detection of an increase and/or fall of cardiac troponin (cTn) with at least one value above the upper reference limit and at least one of the following: (1) symptoms of myocardial ischemia; (2) new ischemic ECG changes or development of pathological Q waves in ECG; (3) imaging evidence of new loss of viable myocardium or new regional wall motion abnormality; (4) identification of an intracoronary thrombus by angiography or autopsy. |
| Arrhythmia | Arrhythmia was defined as any newly diagnosed supraventricular or ventricular rhythm disturbance, including atrial fibrillation, atrial flutter, supraventricular tachycardia, ventricular tachycardia, ventricular fibrillation, or clinically significant bradyarrhythmia that required therapeutic intervention. |
| Heart failure | Heart failure (HF) was defined as an event that met all of the following criteria: (1) The patients had new or worsening symptoms, including at least one of the following: a. dyspnea, b. decreased exercise tolerance, c. fatigue and d. other symptoms of worsened end-organ perfusion or volume overload; (2) The patients had objective evidence of new or worsening CHF, consisting of at least two physical examination findings or one physical examination finding and at least one laboratory/imaging/hemodynamic criterion. a. Physical examination finding: i. Peripheral edema, ii. Increasing abdominal distention or ascites (in the absence of primary hepatic disease), iii. Pulmonary rales/crackles/crepitations, iv. Increased jugular venous pressure and /or hepatojugular reflux, v. S3 gallop, vi. Clinically significant or rapid weight gain thought to be related to fluid retention. b. Laboratory, imaging or hemodynamic evidence of new or worsening CHF, including: i. Increased B-type natriuretic peptide (BNP)/N-terminal pro-BNP (NT-pro-BNP) concentrations consistent with decompensation of heart failure, ii. Radiological evidence of pulmonary congestion, iii. Hemodynamic evidence from right heart catheterization. (3). The patients received initiation or intensification of treatment specifically for CHF, including at least one of the following: a. Significant augmentation in oral diuretic therapy; b. Intravenous diuretic, inotrope, or vasodilator therapy; c. Mechanical or surgical intervention. |
| Stroke | Stroke was defined as an acute episode of focal or global neurological dysfunction caused by central nervous system (CNS) vascular injury as a result of hemorrhage or infarction. Stroke was classified as ischemic stroke and cerebral hemorrhages. Ischemic stroke was defined as an acute episode of focal cerebral, spinal, or retinal dysfunction caused by CNS infarction. Ischemic stroke was defined as an acute episode of focal cerebral, spinal, or retinal dysfunction caused by CNS infarction. |

Abbreviations: BNP, B-type natriuretic peptide; CHF, chronic heart failure; cTn, cardiac troponin; CNS, central nervous system; ECG, electrocardiograph; HF, heart failure; MAP, mean artery pressure.
